# Mixotrophic Cultivation of *Arthrospira platensis* (Spirulina) under Salt Stress: Effect on Biomass Composition, FAME Profile and Phycocyanin Content

**DOI:** 10.3390/md22090381

**Published:** 2024-08-24

**Authors:** Nicola Pio Russo, Marika Ballotta, Luca Usai, Serenella Torre, Maurizio Giordano, Giacomo Fais, Mattia Casula, Debora Dessì, Paola Nieri, Eya Damergi, Giovanni Antonio Lutzu, Alessandro Concas

**Affiliations:** 1 Department of Life Sciences, University of Modena and Reggio Emilia, Via Giuseppe Campi 287, 41123 Modena, MO, Italy; nicolarussopio01@gmail.com (N.P.R.); ballottamarika@gmail.com (M.B.); 2 Teregroup Srl, Via David Livingstone 37, 41123 Modena, MO, Italy; luca.usai@teregrpoup.net; 3 Department of Pharmacy, University of Pisa, Via Bonanno Pisano 12, 56126 Pisa, PI, Italy; serenella.torre@phd.unipi.it (S.T.); paola.nieri@unipi.it (P.N.); 4 Check Lab, Via Acquasanta 16, 84131 Salerno, SA, Italy; giordano.maurizio70@gmail.com; 5 Department of Mechanical, Chemical and Materials Engineering, University of Cagliari, Piazza d’Armi, 09123 Cagliari, CA, Italy; giacomo.fais@unica.it (G.F.); mattia.casula@unica.it (M.C.); 6 Interdepartmental Center of Environmental Science and Engineering (CINSA), University of Cagliari, Via San Giorgio 12, 09124 Cagliari, CA, Italy; 7 Department of Life and Environmental Sciences, University of Cagliari, Cittadella Universitaria, Blocco A, SP8 Km 0.700, 09042 Monserrato, CA, Italy; deboradessi95@gmail.com; 8 Algaltek SARL, R&D Departments, Route de la Petite-Glane 26, 1566 Saint Aubin, FR, Switzerland; eya.damergi@algatek.com

**Keywords:** brewery wastewater, circular bio-economy, *Arthrospira platensis*, salt stress, Phycocianin, FAME profile

## Abstract

*Arthrospira platensis* holds promise for biotechnological applications due to its rapid growth and ability to produce valuable bioactive compounds like phycocyanin (PC). This study explores the impact of salinity and brewery wastewater (BWW) on the mixotrophic cultivation of *A. platensis*. Utilizing BWW as an organic carbon source and seawater (SW) for salt stress, we aim to optimize PC production and biomass composition. Under mixotrophic conditions with 2% BWW and SW, *A. platensis* showed enhanced biomass productivity, reaching a maximum of 3.70 g L^−1^ and significant increases in PC concentration. This study also observed changes in biochemical composition, with elevated protein and carbohydrate levels under salt stress that mimics the use of seawater. Mixotrophic cultivation with BWW and SW also influenced the FAME profile, enhancing the content of C16:0 and C18:1 FAMES. The purity (EP of 1.15) and yield (100 mg g^−1^) of PC were notably higher in mixotrophic cultures, indicating the potential for commercial applications in food, cosmetics, and pharmaceuticals. This research underscores the benefits of integrating the use of saline water with waste valorization in microalgae cultivation, promoting sustainability and economic efficiency in biotechnological processes.

## 1. Introduction

Microalgae represent a promising frontier in biotechnology due to their rapid growth rates, high biomass productivity, and ability to produce a wide array of bioactive compounds [1,2]. These microorganisms can be exploited for various applications, such as biofuel production, wastewater (WW) treatment, CO_2_ sequestration, and the generation of nutraceuticals and pharmaceuticals [3,4,5]. Their ability to thrive in various and harsh environmental conditions makes them versatile candidates for sustainable biotechnological processes [6,7]. Cyanobacteria can produce a large set of valuable compounds [8]. Among them, phycocyanin (PC) is a blue pigment-protein complex which has garnered significant interest due to its extensive applications in the food, cosmetic, and pharmaceutical industries [9]. Indeed, PC is of interest for its antioxidant, anti-inflammatory, and neuroprotective properties and is used for the production of a drug called phycocyanobilin [10]. The filamentous cyanobacterium *A. platensis* (universally known as *Spirulina*)*,* beside its high protein content, productivity and resilience, is also renowned for accumulating high PC levels [11]. Among the various cultivation strategies employed to optimize biomass and metabolite production, mixotrophy stands out as an effective approach [12,13]. Mixotrophy combines autotrophic (photosynthesis) and heterotrophic (organic carbon utilization) modes, potentially enhancing the yield of PC by providing a flexible nutrient environment [14]. One of the most promising aspects of mixotrophic cultivation is the use of organic sources derived from food industry wastes to sustain growth [15]. Dairy wastewater (DWW), rich in lactose and other organic compounds, provides a viable nutrient source for *A. platensis*, promoting biomass and PC production while simultaneously addressing waste management issues [16]. Brewery wastewater (BWW), containing residual sugars and proteins, can similarly support mixotrophic growth, enhancing the economic and environmental sustainability of both the microalgae cultivation and brewing industries [17]. Molasses, a byproduct of sugar processing, offers another potent carbon source, rich in sucrose and other nutrients, that can be utilized to boost PC yield in *Spirulina* cultures [18,19]. Using these food industry wastes not only reduces the cost of cultivation by substituting expensive chemical nutrients but also mitigates environmental pollution by recycling organic waste streams. This synergy between waste valorization and microalgae cultivation promotes sustainability when producing strategic molecules such as PC [16]. Salinity is a crucial environmental factor that significantly influences the physiological and biochemical processes in *A. platensis* metabolism. It affects osmoregulation, enzyme activity, and the structural integrity of cellular components [20]. Under mixotrophic conditions, the interaction between salinity and nutrient availability can further complicate these effects. Salinity stress induces the synthesis of compatible solutes and stress proteins, which can alter the metabolic flux towards secondary metabolite production, including PC [21]. Studies have shown that moderate salinity levels can stimulate the production of PC by enhancing the expression of genes involved in its biosynthesis. However, excessive salinity may lead to osmotic stress, reducing cell viability and PC yield [22]. The balance between salinity levels and nutrient supply is thus critical in optimizing PC production under mixotrophic conditions [23]. Understanding how different waste-derived organic sources interact with salinity levels under mixotrophic conditions will be key to optimizing the sustainable production of PC. This study aims to elucidate the specific impacts of salinity levels on the production of PC by *A. platensis* under mixotrophic conditions in a continuous mode. A second aim is to assess the effects of seawater (SW) on the biochemical composition of *A. platensis* in terms of lipids, proteins, and carbohydrates in view of its use in the food market. To the best of our knowledge, the combined effect of SW and brewery wastewater (BWW) on the content and composition of fatty acids (FAs) is unknown. Therefore, BWW is used to guarantee mixotrophic conditions while cultivating *A. platensis* using SW to assure salt stress. By exploring the biochemical response of *A. platensis* to salinity under mixotrophy, this research seeks to identify optimal cultivation parameters that enhance biomass composition and PC yield.

## 2. Results

### 2.1. BWW Characterization

Table 1 presents the fundamental characteristics of the clarified BWW utilized in this work in comparison with those of other studies. It can be inferred that the loads of organic matter and N in the form of ammonium were consistent compared to other reported BWWs, with total organic carbon (TOC) > 11 g L^−1^ and NH_4_^+^ > 15 mg L^−1^, respectively. It should be considered that, despite the fact that brewery effluents may exhibit different chemical compositions, based on the technological steps employed for manufacturing beer (mashing, boiling, fermentation and maturation, wort separation, wort clarification, and rough beer clarification), these effluents are usually characterized by the presence of easily biodegradable sugars, soluble starch, ethanol, and volatile fatty acids [24]. Their typical high organic content is confirmed by biological oxygen demand (BOD_5_), chemical oxygen demand (COD), and a total suspended solids (TSS) range of 1.2–3.6 g L^−1^, 2–6 g L^−1^, and 0.2–1 g L^−1^, respectively [25]. Usually, BWW is not toxic and is characterized by very low levels of heavy metal as well as N and P levels, which can vary depending on the handling of the raw material used for beer production. The BWW temperature can range from 18 to 40 °C, occasionally reaching values higher than this range [25]. The pH levels, ranging from 4.5 to 12, are highly dependent on the cleaning and sanitizing chemicals used [26]. A common procedure to allow microalgae growth inside a culture medium with a huge organic content, such as for instance a BWW, is its physical and chemical pre-treatment [24]. Due to several chemical and biochemical reactions and several solid–liquid separations performed for beer production, BWW pre-treatment is mandatory. This allows us to reduce and separate the large amounts and different varieties of wastes produced including water, spent grains and trub, hops, caustic and acid cleaners, surplus yeast, waste labels, and waste beer [27].

The choice of using very low concentrations of BWW (2% v v^−1^) was dictated by the elevated TOC of the effluent (Table 1). Previous studies have suggested that the optimal BWW concentration for mixotrophic cultivation of microalgae is less than 3% v v^−1^, with higher concentrations (>30% v v^−1^) resulting in growth inhibition [32]. This was recently confirmed by Miotti et al. [33], who supplemented a mix of synthetic medium and BWW with lower organic carbon content in terms of glycerol, ranging from 0.2% to 2% v v^−1^, to monitor the growth of *Chlorella vulgaris*. 

Additionally, when assessing the N:P molar ratio for the BWW used in this study, considering N (nitrate and ammonia) and P (phosphate) values, it appears to be much lower than the N:P ratios of approximately 10:1 reported in Table 1 for other BWWs by other studies. Moreover, the N:P ratio is smaller than the Redfield ratio (N:P of 16:1), suggesting that the BWW serves as an N-limited medium for microalgae.

### 2.2. Growth Profile and Biomass Composition of A. platensis in BWW and SW

*A. platensis* was cultivated in both photoautotrophic and mixotrophic conditions utilizing brewery effluent (JB) and SW (JBS) at a concentration of 2% v v^−1^ for a duration of 15 days until the early stationary phase of growth was achieved. This investigation aimed to assess key kinetic parameters, including maximum biomass concentration (X_max_), average biomass productivity (∆X), doubling time (t_d_), and specific growth rate (µ).

**Figure 1:**
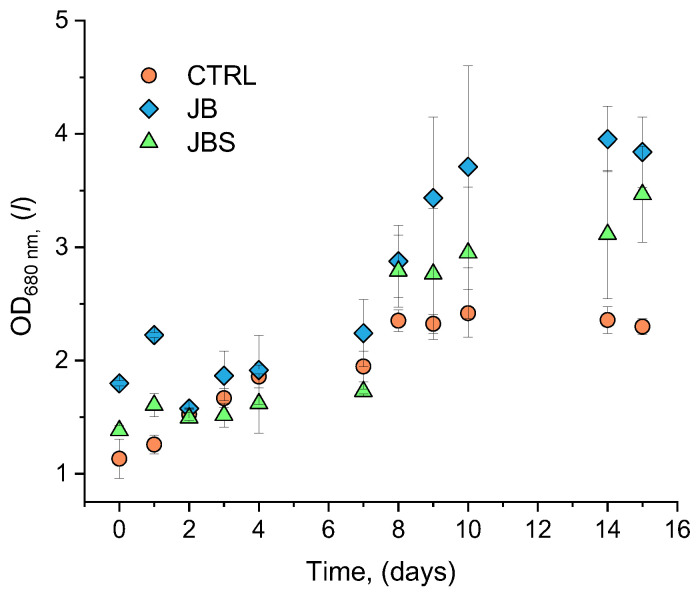
Kinetic evolution of optical density at 680 nm in the investigated cultures.

Figure 1 depicts the progression of *A. platensis* growth curves under both mixotrophic and photoautotrophic conditions. The lag phase persisted for 96 h in both JB and JBS systems while in the control there was no lag phase at all. This was primarily due to the time needed by *A. platensis* to adapt to the new growth conditions represented by the addition of the brewery and SW to the JM. Similar behavior was also reported when cultivating *A. platensis* in both freshwater and SW [34]. At the end of the batch phase, maximum biomass concentrations of 0.98 g L^−1^ and 0.90 g L^−1^ were obtained in freshwater and SW, respectively. In addition, the duration of the batch phase was affected since 9 and 12 days were required to reach the maximum concentration in the two systems. 

In our study, the longer adaptation phase was primarily due to the time needed by *A. platensis* to acclimate to the novel growth conditions represented by the addition of both BWW and SW to the JM. Acclimation is a key phase in cyanobacteria adaptation affecting the overall performance of the culture. Subsequently, the exponential growth phase in all samples persisted for up to 15 days, displaying varying patterns. Around the midpoint of the cultivation period (7 days), all systems exhibited an OD_680nm_ > 1.5. By the end of the cultivation period, the JB and JBS systems outperformed the control, with the JBS system still increasing OD values on the 16th day. 

**Figure 2:**
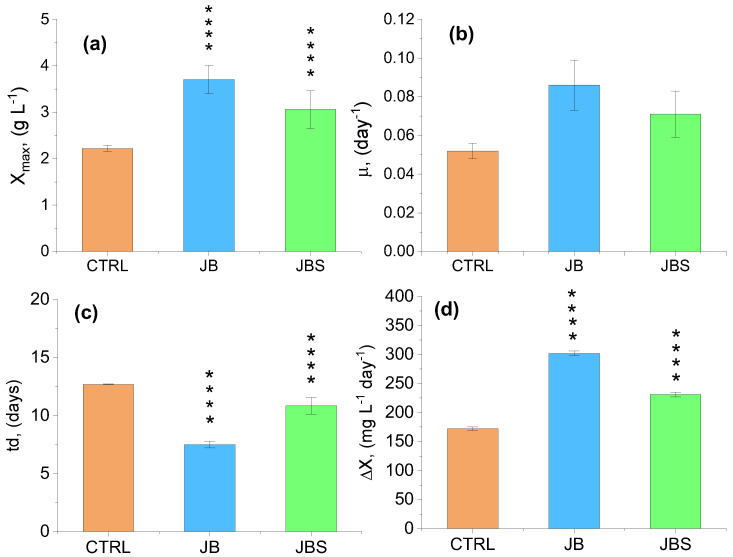
Comparison of growth performance indicators including maximum biomass content *X_max_* (**a**), specific growth rate *µ* (**b**), doubling time *t_d_* (**c**), and average biomass productivity ∆*X* (**d**) for the three investigated media. Mean differences were compared using 2-way ANOVA (*n* = 3, **** indicates *p* < 0.0001).

The maximum biomass production for JB and JBS cultures occurred at the end of the stationary growth phase, while the control was attained after 10 days. The control achieved a maximum concentration of biomass equal to 2.22 g L^−1^, i.e., the lowest among the investigated mixotrophic conditions (Figure 2a).

Remarkably, the photoautotrophic *X_max_* value was 2.5–5 times higher than the values (0.55–0.89 g L^−1^) reported for *A. platensis* by Sassano et al. [35] for the same strain grown in continuous mode using NH_4_Cl as N source. The mixotrophic cultivation of *S*. *platensis* in JBS exhibited the highest biomass yield of 3.70 g L^−1^. In Figure 2b, it is apparent that the JB and JBS systems displayed a superior specific growth rate (0.083 and 0.073 day, respectively) compared to the control (0.055 day). Additionally, JB also showed the highest average biomass productivity at 302 mg L^−1^ day^−1^. This represented about a 50% increase in biomass compared to the control (172 mg L^−1^ day^−1^), as illustrated in Figure 2d. Mixotrophic cultures exhibit elevated growth rates compared to photoautotrophic and heterotrophic cultures. Their ability to assimilate both growth substrates, coupled with the advantage of performing photosynthesis, provides independence. This is advantageous because the acetyl-CoA pool is preserved for both CO_2_ fixation through the Calvin cycle and extracellular organic carbon production [36]. The suitability of BWW to boost mixotrophic metabolism in microalgae and cyanobacteria has been addressed in many scientific reports. Miotti et al. cultivated *Chlorella vulgaris* in BWW with varying concentrations of glycerol under both autotrophic, heterotrophic, and mixotrophic conditions [33]. When *C. vulgaris* was cultivated under mixotrophic conditions, it exhibited a significantly higher biomass yield (1.33 g L^−1^) compared to the autotrophic cultivation (1.08 g L^−1^). The FAME profile analysis revealed that, in comparison to the autotrophic control, higher PUFA contents were obtained under mixotrophy within the range of glycerol mL tested. Similarly, our study observed that mixotrophic cultivation of *A. platensis* in JBS yielded the highest biomass concentration (3.70 g L⁻¹), which significantly surpassed the control. This outcome underscores the potential of mixotrophic systems to enhance biomass productivity, corroborating Miotti et al.’s findings. Moreover, their observation of higher PUFA content under mixotrophic conditions aligns with the increased biomass productivity and superior specific growth rates we reported in the JB and JBS systems. BWW allowed the vigorous growth of a filamentous microalga, *Tribonema aequale*, to a final biomass concentration as high as 6.45 g L^−1^. 

The resulting algal biomass obtained under mixotrophy contained high amounts of high-added value products such as chrysolaminarin, palmitoleic acid, and eicosapentaenoic acid (EPA) [31]. *A. platensis* was also successfully cultivated in non-diluted and non-pretreated BWW under non-sterile and alkaline growth conditions. The mixotrophic growth of this cyanobacterium was evaluated in terms of pollutant consumption, biomass productivity, and composition as well as pigment production. It was obtained that the combination of sodium chloride with sodium bicarbonate determined a maximum final pigment production. Moreover, the use of a photoperiod of 16:8 h caused an increase in the pollutant removal rate (up to 90% of initial concentrations) and biomass concentration (950 mg L^−1^) [37]. Their findings, in terms of biomass productivity, pigment production, and pollutant removal, are indeed noteworthy. In comparison, our research, which utilized a diluted BWW blend, also demonstrated significant improvements in biomass production and *µ* (Figure 2b) under mixotrophic conditions. However, the use of non-diluted BWW in their study suggests the possibility of further optimizing our culture conditions to potentially achieve even higher yields. This comparison highlights the importance of exploring a range of BWW concentrations to balance the benefits of increased organic load with the risks of contamination and growth inhibition.

**Figure 3:**
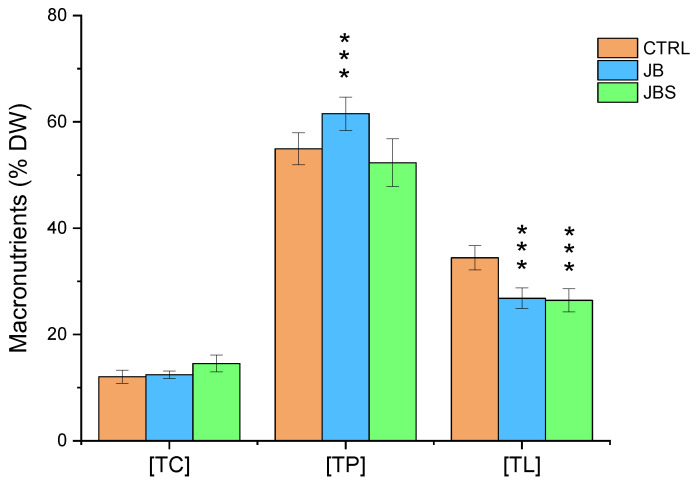
Total carbohydrates (TC), lipids (TL), and proteins (TP) obtained with the investigated media. Mean differences were compared using Tukey’s test (*n* = 3, *** indicates *p* < 0.001).

The effect of mixotrophy on the biomass composition of *A. platensis* in terms of macronutrients, such as total carbohydrates (TC), total proteins (TP), and total lipids (TP), is summarized in Figure 3. 

TP represented the main fraction, followed by TL and TC, both under photoautothropy and mixotrophy. These macronutrients’ distribution, characterized by a remarkable fraction of lipids, differs significantly from a typical chemical composition reported for *A. platensis* which consists of 15–25% carbohydrates, 55–70% proteins, and 4–7% lipids according to Bezerra et al. [38]. Batch or continuous growth impacts not only microalgae growth and biomass productivities but also their biochemical composition [39]. Compared to photoautothropy, there was a considerable rearrangement of proteins and lipids under mixotrophy, with an increase in TP and a decrease in TL after BWW addition. The addition of SW to the JB under mixotrophy produced a considerable decrease in TP with an enhancement of TC fraction. 

The effect of salt on the biochemical composition of biomass is not well understood. Some studies have demonstrated a direct relationship between cultivating *A. platensis* in SW and a reduction in protein content and FAs [34] along with an increased accumulation of carbohydrates [39], essential amino acids including valine, leucine, and isoleucine as well as carotenoids [34]. In another study, a direct relationship between increased salinity and the enhancement of lipid fraction was demonstrated in *Spirulina* [40]. These findings are corroborated by this study in terms of TP, TL, and TC modifications. Salinity is known to influence the morphology of trichomes and the biochemical composition of biomass, making it a potential method for manipulating the morphological and biochemical properties of produced biomass. These effects are obtained after cell acclimation to increasing salinity. However, there are only a few studies on the impact of salt stress after cell acclimation on the biochemical composition and the growth rate of *A. platensis* [21]. In mixotrophic cultivation, several approaches can be applied to improve the production of high-value metabolites. Among them, salinity (along with pH and nutrient availability) as well as the selection of the operation mode (batch, fed-batch, and continuous cultivation) are regarded as valid bioengineering approaches that can be used to optimize microalgae biomass production and composition [41]. It should be noted that not all the microalgae strains adapt to the high salinity levels typical of SW. However, *A. platensis* is known to thrive at the SW salinity level (35 g L^−1^), and the strain used in this study exhibited a tolerance of up to 41 g L^−1^. Studies confirm that biomass production can be enhanced in salinity up to 40 g L^−1^ NaCl, while at 60 g L^−1^ NaCl biomass production decreases slightly [21]. Other authors demonstrated that it is possible to produce *A. platensis* biomass using SW after an adaptation period producing a biomass suitable for food applications [34]. 

### 2.3. Phycobiliproteins Production by A. platensis under Mixotrophy and Salt Stress

**Figure 4:**
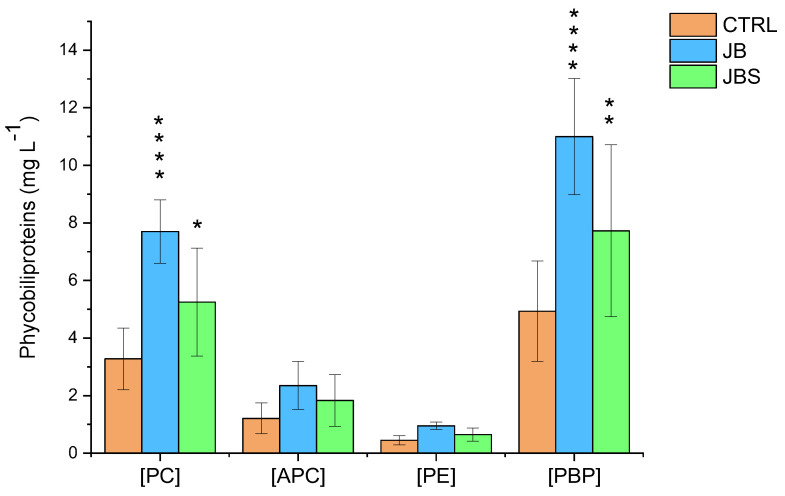
Phycobiliproteins concentration in extracts of *A. platensis* grown in the investigated media. Mean differences were compared using Tukey’s test (*n* = 3, * *p* < 0.05; ** *p* < 0.01; **** indicates *p* < 0.0001).

Figure 4 depicts the concentration of PC, APC, PE, and total phycobiliproteins (PBPs) in extracts from *A. platensis* biomass cultivated both under photoautotrophic and mixotrophic conditions with the addition of BWW (JB) and SW (JBS). 

The analysis revealed that pigment concentrations, especially under mixotrophic conditions with BWW, were particularly relevant. Under these conditions, *A. platensis* showed a notable increase in PC production, more than doubling the concentration in JB and nearly doubling it in JBS when compared to the control (7.70 mg L^−1^ and 5.25 mg L^−1^ vs. 3.28 mg L^−1^). This increase highlights the beneficial effects of mixotrophy on both microalgal growth rates and pigment synthesis, with notably higher levels of PC and APC compared to the control. 

Previous research suggests that the increase in PC production is influenced by a complex interaction between culture medium composition, the presence of an organic carbon source, and the physiological responses of microalgae to specific culture conditions, which create a stress environment favorable for PC synthesis. 

In this study, it was evident that mixotrophic cultures had a significant increase in overall PBP concentration compared to the photoautotrophic control. Specifically, JB showed the highest total PC content at 7.70 mg L^−1^, confirming the effectiveness of BWW supplementation in enhancing pigment synthesis. Similarly, APC and PE concentrations increased significantly, reaching 2.35 mg L^−1^ and 0.95 mg L^−1^, respectively, compared to the control values of 1.21 mg L^−1^ and 0.45 mg L^−1^. 

These findings are consistent with the trend observed during the mixotrophic growth of *A. platensis* using cheese whey as an organic carbon source. In this case, the positive correlation between organic load and increased PBP content was demonstrated in an upscaled volume process [16]. Similarly, higher PC content compared to the conventional control Zarrouk medium was reported for *A. platensis* cultivated mixotrophically in tofu WW [42], and for the microalga *Galdieria sulphuraria* ACUF 064 grown in media containing buttermilk as a carbon source [43]. Regarding the correlation between salinity and PBP production, the addition of SW to JB resulted in a slight increase in PC, APC, and PE. These results are consistent with documented behavior in *A. platensis*, which exhibited increased protein and PC content when different NaCl concentrations were added [21,40]. 

The suitability of PC for various applications largely depends on its purity, which is typically assessed using an absorbance ratio. This ratio measures PC’s absorbance at 620 nm (A620) against that of other proteins at 280 nm (A280). A PC with an A620/A280 ratio, defined as extraction purity (EP), of 0.7 or higher is classified as food grade, making it apt for use as a food additive or a natural blue pigment in cosmetics. When EP falls between 0.7 and 3.9, PC is considered a reagent grade, with EP values of 1.5 or higher suitable for cosmetic applications. An EP of 4 or more qualifies PC as an analytical grade, which is suitable for pharmaceutical uses [44].

**Figure 5:**
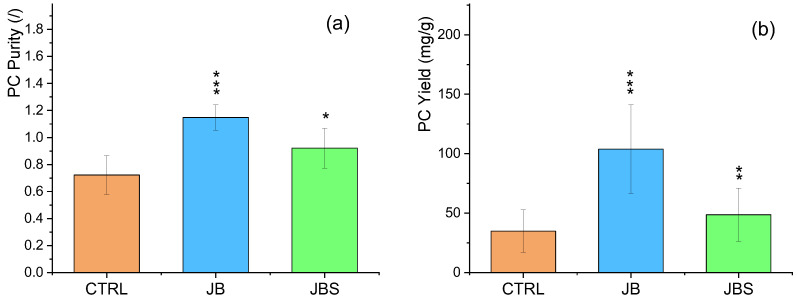
Purity (**a**) and yield (**b**) of PC in the investigated media. Mean differences were compared using 2-way Tukey’s test (*n* = 6, * *p* < 0.05; ** *p* < 0.01; *** indicates *p* < 0.001).

In the current study, PC purity under mixotrophy conditions was found to have an EP of 1.15 with JB and 0.92 with JBS, compared to a control of 0.77 (Figure 5a). Correspondingly, the PC yields were 100, 50, and 35 (mg g^−1^) for JB, JBS, and CTRL cultures, respectively (Figure 5b). These findings are consistent with previous research by Khandual et al. [45] on *A. platensis* and by Chini Zittelli et al. [46] on various strains, which demonstrate that an organic source (such as dairy WW or BWW) in concentrations of 0.5–2% v v^−1^ can enhance PC synthesis in mixotrophic cultures of *A. platensis*.

PC purity is closely linked to the extraction methods used, which are influenced by physical and chemical parameters such as temperature, pH, solvent type, biomass-to-solvent ratio, and the form of biomass (dried or fresh). The commercial value of PC varies greatly depending on its purity level. According to the literature, the cost of PC increases with its purity, particularly in industries like cosmetics, agro-chemistry, and food [47]. For instance, analytical-grade PC with a purity exceeding 4 can cost up to 60 US$ g^−1^, and for the highest purity levels, prices can soar up to 19,500 US$ g^−1^. Recent studies indicated that PC for biocolorant use costs around 0.35 US$ g^−1^, while analytical-grade PC can reach up to 4500 US$ g^−1^ [48]. The global PC market is projected to grow to $245.5 million by 2027 and $279.6 million by 2030 [49]. These high prices are due to the challenges in the extraction and purification processes, making PC an expensive protein pigment. Overall, the findings from this study on PC yield and purity offer new insights into developing algal cultivation strategies that maximize the production of bioactive components while promoting sustainable and eco-friendly practices. 

The increased yield of PC observed in JB and JBS compared to the control may be due to the cellular stress from the abrupt shift from autotrophic to mixotrophic conditions and the high turbidity and salinity of BWW. BWW is known for its turbidity and salinity [50]. When microalgae are transferred to a more opaque medium, lower light intensity might inhibit photosynthesis, promoting cells to overexpress light-harvesting pigments like PC to capture light more efficiently [51]. 

Conversely, high light intensity can downregulate PC content to avoid excessive electron formation and oxidative damage [52]. High PC content in some strains is often observed under very low light intensity [53,54]. Using continuous cultivation modes can mitigate excessive turbidity, enhancing medium transparency and thereby potentially increasing PC content [55]. Therefore, the increased PC content observed when passing from the control to BWW and SW-supplemented media could be ascribed to this phenomenon. Additionally, osmotic stress can lead specific microalgae strains to boost PBP production [22]. High salinity may cause rapid sodium ion absorption, leading to phycobilisome separation from thylakoid membranes [56]. Cells responding to increased salinity may overregulate PC production to counteract this effect. Thus, the high salinity of BWW likely contributed to the increased PC observed in microalgae cultivated under mixotrophic conditions in JB and especially in JBS.

### 2.4. FAME Profile by A. platensis under Mixotrophy

The fatty acid methyl ester (FAME) profile of *A. platensis* under mixotrophic conditions with (JBS) and without (JB) salt stress, compared to the control (CTRL), is shown in Table 2. Overall, there were no significant differences in the FAME profile between the photoautotrophic and mixotrophic cultures systems, except for certain FAs. Specifically, myristic acid (C14:0), palmitic acid (C16:0), and oleic acid (C18:1) exhibited higher levels under mixotrophy, whereas stearic acid (C18:0), linoleic acid (C18:2), and y-linolenic acid *n*-6 (C18:3) had lower values. In CTRL, the most abundant FA was C16:0 (40.57%), followed by C18:3 (15.59%), C18:2 (15.18%), and stearic acid C18:0 (15.6%). In the JB group, C16:0 was also the most prevalent (43.77%), with C18:0 (12.73%), C18:2 (12.47%), and C18:3 *n*-6 (12.27%). For the JBS group, the sequence of FAs’ predominance resulted in C16:0 (41.48%) > C18:2 (14.68%) > C18:3 *n*-6 (14.57%) > C18:0 (13.43).

The C16:0 was the predominant FA across all the cultivation systems. The total percentages of C16–C18 FAs in *A. platensis* showed minor differences between photoautotrophic (96.02%) and mixotrophic conditions (94.56% and 95.28%). These findings are consistent with the study by Cavallini et al. [16], who observed a similar FA distribution pattern in *A. platensis* cultivated under both autotrophy and mixotrophy conditions with cheese whey supplementation. This study also found no significant changes in the levels of saturated (SFAs), monounsaturated (MUFAs), and polyunsaturated fatty acids (PUFAs) when *A. platensis* was grown under photoautotrophy (CTRL) and mixotrophy with salt stress (JBS). However, mixotrophy without salt stress (JB) resulted in increased levels of SFAs and MUFAs and a decrease in the total unsaturated fatty acids (UFAs) and PUFAs.

While C16:0 is an important energy source in infant nutrition, given its presence in breast milk at levels between 20 and 30%, heightened levels of free SFAs, including C16:0 and, to a lesser extent, C18:0, are correlated with an elevated risk of cardiovascular disease in adults [57]. This association is primarily due to vascular endothelial dysfunctions linked to oxidative stress, which arises from increased mitochondrial uncoupling [58]. Medical and pharmaceutical research highlights the importance of maintaining normal or non-elevated levels of C16:0 and C18:0 within the physiological range to prevent oxidative stress in the vascular endothelium. The FAME profile observed in this study is like that of olive oil, containing beneficial MUFAs known for their positive effects in preventing cardiovascular disease and addressing various risk factors. Therefore, it can be hypothesized that regular consumption of *A. platensis* biomass, where the levels of these two FAs do not exceed physiological limits, as seen in CTRL, JB, and JBS, could play a significant role in protecting cells against endothelial dysfunctions [58].

The α-linolenic acid (ALA, C18:3 *ω*-3) and γ-linolenic acid (GLA, C18:3 *ω*-6) are two PUFAs commonly found in microalgal and cyanobacterial oils [59]. Consistent with our results, various scientific reports have shown that GLA is the predominant isomer of this FA synthesized by *A. platensis* [60]. A recent review by [61] discussed several culture conditions (temperature, light intensity, nitrogen cell concentration, growth phase, and light/dark cycle) that enhance lipids and GLA production in *Spirulina*. It was noted that both lipid content and GLA levels are higher under mixotrophy compared to autotrophic conditions. However, our study did not align with this finding, as the addition of BWW to the culture medium reduced GLA content from 15.59% under photoautotrophy (CTRL) to 12.27% under mixotrophy (JB), with the addition of salt to JB only slightly decreasing the GLA content to 14.57%.

The content of ALA in *A. platensis* is generally negligible compared to GLA. In our study, ALA content increased significantly with the addition of BWW, rising from 1.19% in the CTRL to 1.43%, while the inclusion of salt led to a substantial decrease to 0.75%. ALA plays a critical biological role, particularly as a precursor for the synthesis of eicosapentaenoic acid C20:5 ω-3 (EPA) and docosahexaenoic acid C22:6 ω-3 (DHA). ALA is one of two essential FAs that humans must obtain through their diet for optimal health because it cannot be synthesized internally. The human body must convert ALA into EPA and DHA to harness its biological benefits, essential for the proper functioning of vital organs. This conversion process, although not highly efficient, allows the body to utilize 5% to 10% of the consumed ALA, with approximately 7% converting into EPA and from this, about 1% further converting into DHA [62].

The ratio of PUFA to SFA is a crucial parameter in assessing the nutritional quality of foods and their impact on cardiovascular health. PUFAs are known to lower low-density lipoprotein cholesterol (LDL-C) and overall serum cholesterol levels, whereas SFAs generally increase cholesterol levels. A higher PUFA:SFA ratio is thus considered beneficial. The British Department of Health recommends a PUFA:SFA ratio exceeding 0.45 in the human diet, and WHO/FAO guidelines suggest a balanced diet should have a PUFA:SFA ratio above 0.4, as the higher ratio is believed to mitigate the risk of cardiovascular and other chronic diseases [63]. In this study, the observed ratio exceeded 0.4 for both *A. platensis* cultivated under both autotrophy (0.85) and mixotrophy with (0.84) and without (0.74) the addition of salt.

The ratio of Σ hypocholesterolemic FAs/Σ hypercholesterolemic FAs (h/H) is another important factor in cholesterol metabolism. Higher h/H values are considered more favorable for human health, as they may accurately reflect the impact on cardiovascular disease. In our study, *A. platensis* showed an h/H ratio of 0.85 under photoautotrophic conditions and 0.74 under mixotrophic conditions with the addition of BWW to JM. When salt was added to JB, the value reverted to that of the control at 0.84. These h/H ratios under mixotrophy were higher than those (0.60–0.66) reported for *Spirulina* sp. by other authors [64,65]. However, various freshwater microalgae strains such as *Scenedesmus obliquus* (2.9), *Chlorella vulgaris* (2.8–2.04), *Porphyridium cruentum* (1.90), *Phaeodactylum tricornutum* (1.8–1.72), *Chlorococcum amblystomatis* (1.7), *Nannochloropsis oceanica* (1.7), *Nannochloropsis oculata* (1.44), and *Tetraselmis chui* (1.4) exhibited higher values [64,65]. It should be noted that variations in oil recovery methods (including the solvents used) from microalgae cells are not consistently specified in the literature.

## 3. Materials and Methods

### 3.1. Inoculums and Culture Media Preparation

The strain *Arthrospira platensis* SAG 21.99 used in this study was sourced not axenic from the culture collection of algae at the Gottingen University, Germany. The cell cultures were maintained and grown in a modified version of the Jourdan Medium (JM), composed as follows: in g L^−1^: 5 NaHCO_3_; 1.6 KOH, 5 NaNO_3_; 0.027 CaCl_2_ * 2H_2_O; 0.4 K_2_SO_4_, 2 K_2_HPO_4_; 1 NaCl; 0.4 MgSO_4_ * 7H_2_O; 0.16 EDTA-Na_2_; 0.01 FeSO_4_ * 7H_2_O; and 1 mL of Trace elements. The Trace elements solution was prepared with the following composition (mg L^−1^): 250 EDTA-Na_2_; 57 H_3_BO_3_; 110 ZnSO_4_ * 7H_2_O; 25.3 MnCl_2_ * 4H_2_O; 8.05 CoCl_2_ * 6H_2_O; 7.85 CuSO_4_ * 5H_2_O; and 5.5 Mo_7_O_24_(NH_4_)_6_ * 4H_2_O. The original version of JM is detailed in Jourdan. Erlenmeyer flasks of 150 mL were filled with 50 mL of JM medium, inoculated with approximately 10 mL of microalgae, sealed with a cotton plug, and continuously illuminated at room temperature by white fluorescent lamps (Model T8 36 W IP20, CMI, Ense-Höingen, Germany) providing a light intensity of 50 µmol m^−2^ s^−1^, measured with a luxmeter (Model HD 2302.0, Delta OHM, Padua, Italy). The inoculum was cultivated for about one week, until the end of exponential growth phase, before being used for experiments. BWW samples were collected from “Birrificio Emiliano”, a brewery in Anzola, BO, Italy. The main chemical–physical parameters of these effluents are presented in Table 1. 

Once collected, BWW was stored at 4 °C until use. It was then filtered using glass filter microfiber disks (GF/CTM 47 mm diameter, Whatman, Incofar Srl, Modena, MO, Italy), to remove solid materials, and sterilized at 121 °C and 0.1 MPa for 20 min before microalgae cultivation. Seawater, collected from the Adriatic Sea (N 42°67′07.58, E 14°02′43.14), was also sterilized at 121 °C and 0.1 MPa for 20 min.

### 3.2. Cultivation Conditions and Experimental Setup

*A. platensis* was cultivated in 20 L cylindrical PVC reactors (hereafter referred to as PBRs) with an outer diameter of 16 cm, inner diameter of 14 cm, and a height of 27 cm. Filtered compressed air, provided by an air pump (GIS Air Compressor, Carpi, MO, Italy), was supplied to the PBRs through a perforated rubber. PBRs were illuminated with a photoperiod of 12 h light/12 h dark at room temperature by four LEDs (model 2835, 30 W IP33, ImportLed, Desio, MB, Italy) providing a warm white light with an intensity of 150 µmol m^−2^s^−1^. A series of experiments were conducted with a working volume of 3 L to evaluate cell growth, biomass composition, FAME profile, and phycobiliproteins’ (PBPs) production of *A. platensis*. In these experiments, JM was used as control (CTRL). In one setup, only BWW was added to JM (JB), and in another, both BWW and SW were added to JM (JBS). The PBRs operated in continuous mode with a daily removal and addition of specific percentages of JM, BWW, and SW according to the experiments setup detailed in Table 3. All tests were carried out at least in triplicate and lasted for 15 days. Microalgae growth was monitored by measuring optical density and biomass concentration. After cultivation, the final dry weight (g L^−1^) and PBP content (mg g^−1^_DW_) were determined. In all experiments, the initial concentration of the inoculum was 0.3 g L^−1^.

### 3.3. Cell Growth and Dry Weight Determination

*A. platensis* growth was evaluated by monitoring the absorbance (ABS) of the culture at 680 nm using a spectrophotometer (model ONDA V30 SCAN—UV VIS, ZetaLab, Padua, Italy). A regression equation was calculated to describe the relationship between dried biomass concentration and ABS. Dry biomass concentration was evaluated gravimetrically as follows: (a) a known volume (10 mL) of culture (*V*) was drawn from the PBRs, (b) the sample was filtered through a pre-weighted (*W_1_*) glass microfiber filter (GF/C^TM^ 55 mm diameter, Whatman, Incofar Srl., Modena, Italy), the biomass retained on the filter was dried at 105 °C overnight to a constant weight (*W*_2_), and (c) the filter paper had been previously dried in a forced-air oven (model 30, Memmert Gmbh, Scwabach, Germany) at 105 °C for 2 h, then cooled to room temperature in a desiccator, and weighed using an analytical scale (model M, Bel Engineering Srl, Monza, MI, Italy). The cell concentration (dry weight), *X* (g L^−1^) was calculated using the following equation:

(1) $$X=\frac{W_{1}-W_{2}}{V}$$

where *W* = weight (g) of dried algal biomass, and *V* = volume (L) of the algae culture used for the test. The average biomass productivity (∆*X*) was expressed as follows:

(2) $$\Delta X=\frac{X_{max}}{t_{max}}$$

where *X_max_* is the maximum biomass (g L^−1^) obtained at (*t_max_*). The specific growth rate (*μ*) was calculated according to the following equation:

(3) $$\mu =\frac{\ln\left(X_{2}-X_{1}\right)}{\left(t_{2}-t_{1}\right)}$$

where *X*_2_ and *X*_1_ correspond to dry biomass concentration (g L^−1^) at time *t*_2_ and *t*_1_, respectively.

The pH of culture suspensions was measured by a pHmeter (model HI 2210, Hanna Instruments, Woonsocket, RI, USA). 

### 3.4. Phycobilinproteins Extraction and Spectrophotometric Determination 

The extraction of PBPs was conducted using an aqueous saline solution as described by [66]. Specifically, a known amount (10 g) of frozen *A. platensis* biomass was placed in 50 mL of a buffer solution consisting of water with 10 g L^−1^ of calcium dichloride (1%), then frozen and thawed repeatedly until the cells were completely broken up. This mixture was stirred for 30 to 45 min. This extraction process was performed twice, and the resulting phycobilins solution was separated by centrifugation at 8000 rpm for 10–15 min. The blue-colored supernatant was recovered and then used for optical readings with a spectrophotometer. The concentration of different PBPs, including C-phycocyanin (*PC*), allophycocyanin (*APC*), and phycoerythrin (*PE*), were determined by measuring the absorbance of each extract at three different wavelengths: 565, 620, and 650 nm. 

The concentration of these *PBPs* was then calculated using the equations established by [67].

(4) $$\;\;\left[PC\right]=\frac{A_{620}-0.72\;A_{652}}{6.29}$$

(5) $$\left[APC\right]=\frac{A_{652}-0.191\;A_{620}}{5.79}$$

(6) $$\left[PE\right]=\frac{A_{565}-2.41*\left[PC\right]-1.40\;\left[APC\right]}{13.02}$$

The total *PBP* concentration was evaluated as the sum of *PE*, *PC*, and *APC* (mg mL^−1^) of the collected supernatant as follows:

(7) $$\left[PBPs\right]=\left[PC\right]+\left[APC\right]+\left[PE\right]$$

The extraction yield, estimated by relating the concentrations to the biomass of *A. platensis* used (expressed in mg of dry weight), was obtained according to Equation (8):

(8) $$PBP\;\left(\frac{mg}{g}\right)=\frac{\left[PBPs\right]\;V_{ext}}{W_{b}.0.1}$$

In this equation, where *V_ext_* (mL) represents the volume of the extract and *W_b_* (g) denotes the weight of the wet biomass subjected to the extraction process, it is assumed based on experimental observations and literature data that the biomass pellet subjected to extraction contains 10% solid content.

The purity of PC was calculated according to the following equation:

(9) $$PC\;purity\;(/)=\frac{\;A_{620}}{A_{280}}$$

### 3.5. FAMEs Determination

The fatty acids methyl ester (FAME) profile analysis in this study followed the method proposed by [68]. The 10 mg of biomass was weighed using a tube of glass after lyophilization. Subsequently, the biomass was suspended in a suitable volume of a methanol/chloroform (4:5 v v^−1^) mixture along with 50 mg L^−1^ of the internal standard tritridecanoin (TAG 39:0, 13:0/13:0/13:0). The samples underwent eight rounds of vortexing for 60 s each, followed by sonication using an ultrasonic bath for 15 min at 5 °C. Subsequently, 2.5 mL of MilliQ water containing Amino-2-hydroxymethyl-propane-1,3-diol (Tris) (50 mg L^−1^) and NaCl (1 M), with a pH adjusted to 7.0 using an HCl solution, were added. The solutions underwent sonication for 10 min at 5 °C. Subsequently, the samples were centrifuged for 10 min at 177 rcf at 5 °C and the chloroform phase was carefully transferred into a glass tube. The remaining samples were subjected to re-extraction with 1 mL of chloroform and were then further sonicated for 10 min at 5 °C. After an additional centrifugation step, the chloroform phase was mixed with the one formerly collected and then dried using a mild stream of nitrogen.

To obtain fatty acid methyl esters (FAMEs), the fatty acids (FAs) underwent trans-esterification. This process involved the addition of 3 mL of methanol, which contained 5% (v v^−1^) sulfuric acid, to the tube containing the dried lipid samples. The mixture was then incubated at 70 °C for a duration of 3 h. After cooling, 3 mL of n-hexane and 3 mL of MilliQ water were added to the samples. The mixtures were vortexed three times for 1 min at 10 min intervals and then centrifuged at 177 RCF (Relative Centrifugal Force) at 5 °C for 10 min. For every sample, 2 mL of the hexane phase was recovered and washed with 2 mL of MilliQ water twice. The hexane phase with the FAMEs was then put into glass vials for subsequent GC-MS analysis. A gas chromatography trace 1300 equipped with a triple quadrupole mass spectrometry (TSQ 9000), a fused capillary column Agilent HP-5 (30 m × 0.32 i.d, 0.25 μm f.t.), and an automatic sampler (AI 1310) with a split-splitless injector were used (Waltham, MA, USA). The injector was set at 250 °C, and the carrier gas (helium) flow was 1.5 mL min^−1^. The oven temperature was initially held at 50 °C for 1 min, followed by an increase from 50 to 175 °C at 10 °C min^−1^. It was then kept at 175 °C for 10 min, augmented from 175 to 210 °C at 5 °C min^−1^, kept at 210 °C for 10 min, and further increased at 5 °C min^−1^ from 210 °C to 230 °C. Afterwards, it was held for 9.5 min at 230 °C and then re-increased at 10 °C min^−1^ from 230 °C to 300 °C. The sample was injected in split mode (0.4 μL) with a split ratio set at 1:20. The mass spectrometry transfer line temperature and ion source were set at 250 °C and 300 °C, respectively. Ions were generated at 70 eV with electron ionization and recorded at 1.5 scans s^−1^ over the mass range m z^−1^ 50 to 550. Peak identification was conducted by comparing peak retention time with Supelco 37 component FAME Mix (Sigma Aldrich). Data are expressed as a mg g^−1^ of dry weight (mean ± standard deviation) and calculated using Equation (10) by [68]:

(10) $$FA\left(\frac{mg}{g}\right)=IS\;\frac{\frac{A_{{FAME}_{i}}}{A_{C13:0}\;{RRF}_{{FAME}_{i}}}}{m_{b}}$$

where *IS* refers to the internal standard added; $A_{{FAME}_{i}}$ represents the area of the *i*th *FAME*; *A*(*C*13:0) is the area of the *FAME C*13:0; ${RRF}_{{FAME}_{i}}$ denotes the relative response factor of the *i*th *FAME*; and $m_{b}$ is the mass of the processed biomass. The relative abundance of the generic *FA* was determined by dividing its concentration by the total *FA* content. The ratio between the sum of hypocholesterolemic *FAs* and the sum of hypercholesterolemic *FAs* (*h*/*H*) was calculated according to the equation suggested by [69]:

(11) $$\frac{h}{H}=\frac{\sum_{j=1}^{n}C_{j}^{hypo}}{\sum_{k=1}^{m}C_{k}^{Hype}}$$

with *j* = 18:1 *n*-9, 18:1 *n*-7, 18:2 *n*-6, 18:3 *n*-6, 18:3 *n*-3, 20:3 *n*-6, 20:4 *n*-6, 20:5 *n*-3, 22:4 *n*-6, 22:5 *n*-3, and 22:6 *n*-3 while k = 14:0 and 16:0.

### 3.6. Statistical Analysis

Each experimental condition was investigated in triplicate. Statistical analysis was carried out using the software MetaboAnalysts 5.0 tuned by McGill University, Montreal, Canada. In particular, the difference among groups was evaluated by the one-way analysis of variance (ANOVA) followed by the honestly significant difference (HSD) test by Tukey. Variations were considered significant at 95% confidence.

## 4. Conclusions

This study highlighted the efficacy of using food industry wastes like BWW, which is rich in organic carbon, as a cost-effective and sustainable nutrient source for *A. platensis*. The application of BWW not only promoted higher biomass yields (3.70 g L^−1^) but also addressed waste management issues by recycling industrial effluents. The mixotrophic conditions facilitated higher PC production, with BWW and SW supplements notably enhancing PC content (7.70 mg L^−1^ and 5.25 mg L^−1^) compared to controls (3.28 mg L^−1^). The observed increase in PC yield was attributed to the synergistic effects of mixotrophy and salt stress, which stimulated the metabolic pathways involved in PC biosynthesis. Additionally, the study explored the impact of salinity on the biochemical composition of *A. platensis* biomass. The addition of SW resulted in significant changes in protein, carbohydrate, and lipid fractions, suggesting that salinity stress can be leveraged to manipulate the biochemical profile of microalgal biomass. This adaptability of *A. platensis* to saline environments underscores its potential for large-scale cultivation in marine or brackish waters, thus expanding its applicability in diverse geographic regions. Mixotrophic cultivation with BWW and SW also influenced the FAME profile, enhancing the content of specific FAs such as C16:0 (43.77% and 42.48%) and C18:1 (7.85% and 6.49%). This modification in the lipid profile could have implications for the nutritional and commercial value of the biomass produced. The purity of the extracted PC under mixotrophic conditions was found to be suitable for various applications, including food and cosmetic industries, with BWW and SW supplementation yielding higher EP values (1.15 with JB and 0.92 with JBS, respectively). The economic analysis of PC production emphasized the potential market value of high-purity PC, which can reach significant commercial prices, highlighting the economic benefits of optimizing cultivation parameters for maximum PC yield. Overall, this study contributes to the understanding of the complex interactions between salinity, organic carbon sources, and *A. platensis* metabolism. It underscores the feasibility of using mixotrophic cultivation strategies to enhance biomass and metabolite production while promoting environmental sustainability through waste valorization. The findings pave the way for developing efficient and cost-effective bioprocesses for the profitable production of high-added-value compounds from microalgae, aligning with the principles of a circular bio-economy. Future research should focus on scaling up these processes and further exploring the biochemical mechanisms underlying the observed enhancements to optimize industrial applications.

## Figures and Tables

**Table 1:** Physiochemical characteristics of brewery wastewater.

Parameter	This Work	[26]	[28]	[29]	[30]	[31]
TSS	-	0.2–1	-	-	-	0.66–0.77
BOD	-	1.2–3.6	-	-	-	-
COD	-	2–6	2.1–5.8	-	2	2.1–3.54
TOC	11.2	25–80 *	-	16.5	-	-
TN	0.427	10–50 *	5 *	1.2	23 *	23–69 *
N-NO_3_^2−^	-	-	-	-	-	0.1–9 *
N-NH_4_^+^	26.55 *	-	0.004 *	11.3	18 *	18–48 *
TP	0.1 *	-	0.44 *	95.2 *	-	8.2–18.4 *
P-PO_4_^3−^	-	-	-	-	-	4–6 *
Fe	0.1 *	-	-	-	-	-
Mg	0.1 *	-	-	-	-	-
pH	6.7	4.5–1.2	6.9	6.1	-	7.66–7.89

Note: TSS = total suspended solid, BOD = biological oxygen demand, COD = chemical organic demand, TOC = total organic carbon. All the concentrations are expressed in terms of g L^−1^, except those with * which are reported in terms of mg L^−1^.

**Table 2:** Effect of different growth media on FAME composition. Values are reported as mean % ± standard deviation (*n* = 6). The percentages refer to the total weight of FAMEs produced.

FAME	C:N ^$^	CTRL	JB	JBS
Myristic acid	14:0	1.98 ± 0.30	3.14 ± 0.40 ****	2.70 ± 0.91 **
Palmitic acid	16:0	40.57 ± 2.97	43.77 ± 4.00	41.48 ± 4.48
Hexadecenoic acid	16:1	4.12 ± 0.62 ****	3.24 ± 0.80	3.20 ± 0.45 ****
Heptadecenoic acid	17:0	0.19 ± 0.06	0.27 ± 0.06	0.18 ± 0.04
cis-10-Heptadecanoic acid	17:1	0.56 ± 0.10	0.61 ± 0.14	0.54 ± 0.09
Stearic acid	18:0	15.16 ± 1.69	12.73 ± 6.14	13.43 ± 4.59
Elaidic acid	18:1	0.71 ± 0.09	0.80 ± 0.34	0.68 ± 0.15
Oleic acid	18:1	3.50 ± 1.65	7.85 ± 2.09	6.49 ± 2.63 *
Linoleic acid	18:2	15.18 ± 1.32	12.47 ± 1.14	14.68 ± 1.61
α-Linolenic acid	18:3 (*ω*-3)	1.19 ± 0.21	1.43 ± 0.43	0.75 ± 0.51
y-Linolenic acid	18:3 (*ω*-6)	15.59 ± 1.49	12.27 ± 2.57	14.57 ± 2.30
8,11,14-Eicosatrienoic acid	20:3	0.87 ± 0.10	1.04 ± 0.08	0.89 ± 0.14
13-Docosenoic acid	22:1	0.39 ± 0.05	0.38 ± 0.09	0.42 ± 0.08
Σ SFAs	/	57.84	59.91	57.79
Σ UFAs	/	42.11	40.09	42.22
Σ MUFAs	/	9.28	12.88	11.33
Σ PUFAs	/	32.83	27.21	30.89
PUFA:SFA	/	0.57	0.45	0.53
C16-C18	/	6.02	94.56	95.28
h/H	/	0.85	0.74	0.84

Note: $, which represents C:N, refers to number of atom carbon (C) and double bonds (N). Mean differences were tested using two-way ANOVA. No asterisk denotes *p*-value > 0.05; * *p*-value < 0.05; ** *p*-value < 0.01; **** *p*-value < 0.0001.

**Table 3:** Experimental setup showing the % (v v^−1^) of BWW and SW added to the control.

	JM	BWW	SW
CTRL	2.5	-	-
JB	2.5	2	-
JBS	2.5	2	2

Note: JM = Jourdan medium, BWW = brewery wastewater, SW = seawater.

## Data Availability

Data will be available upon request.
